# Expression of ABCA1 Transporter and LXRA/LXRB Receptors in Placenta of Women with Late Onset Preeclampsia

**DOI:** 10.3390/jcm11164809

**Published:** 2022-08-17

**Authors:** Hubert Wolski, Marcin Ożarowski, Grażyna Kurzawińska, Anna Bogacz, Marlena Wolek, Małgorzata Łuszczyńska, Krzysztof Drews, Aleksandra E. Mrozikiewicz, Przemysław Ł. Mikołajczak, Radosław Kujawski, Bogusław Czerny, Tomasz M. Karpiński, Agnieszka Seremak-Mrozikiewicz

**Affiliations:** 1 Division of Perinatology and Women’s Disease, Poznań University of Medical Sciences, 61-701 Poznan, Poland; 2 Division of Obstetrics and Gynecology, Poviat Hospital, 34-500 Zakopane, Poland; 3 Department of Biotechnology, Institute of Natural Fibres and Medicinal Plants, 60-630 Poznan, Poland; 4 Laboratory of Molecular Biology, Division of Perinatology and Women’s Diseases, Poznań University of Medical Sciences, 61-701 Poznan, Poland; 5 Department of Stem Cells and Regenerative Medicine, Institute of Natural Fibres and Medicinal Plants, 60-630 Poznan, Poland; 6 Department of Infertility and Reproductive Endocrinology, Poznań University of Medical Sciences, 61-701 Poznan, Poland; 7 Department of Pharmacology, Poznań University of Medical Sciences, 61-701 Poznan, Poland; 8 Department of General Pharmacology and Pharmacoeconomics, Pomeranian Medical University, 70-204 Szczecin, Poland; 9 Department of Medical Microbiology, Poznań University of Medical Sciences, 61-701 Poznan, Poland

**Keywords:** preeclampsia (PE), adenosine triphosphate-binding cassette transporter A1 (ABCA1), liver X receptors (LXRs), gene expression, protein level

## Abstract

Background: Appropriate levels of cholesterol are necessary for the mother and developing fetus, but theirexcess may cause preeclampsia. The ABCA1 transporter mediates the secretion of cholesterol and is highly regulated at the transcriptional level via the nuclear liver X receptors (LXRs). Methods: Sixteen preeclamptic and 39 normotensives healthy women with uncomplicated pregnancies were involved in the case-control study. The placental levels of ABCA1, LXRA and LXRB mRNA were quantified by real-time quantitative PCR. The concentrations of ABCA1, LXRA and LXRB proteins from the placenta were determined using an enzyme-linked immunosorbent assay Results: We found in the logistic regression model significantly lower placental expression of LXRB mRNA (crude OR = 0.26, 95% CI: 0.07–0.94, *p* = 0.040) and LXRA protein level (crude OR = 0.19, 95% CI: 0.05–0.69, *p* = 0.012) in late-onset preeclamptic women compared to healthy pregnant women. The values remained statistically significant after adjustment for possible confounders. Conclusions: Our results suggest that high placenta LXRA mRNA and LXRA protein expression levels decrease the risk of late-onset preeclampsia. These nuclear receptors could play a role in the development of preeclampsia through disturbances of lipid metabolism.

## 1. Introduction

Preeclampsia (PE) is a multifactorial disease with high blood pressure and proteinuria. This condition complicates 3–8% of all pregnancies worldwide and is the main cause of increased maternal and fetal mortality [1]. New epidemiological estimates suggest that hypertension during pregnancy causes 14% of all maternal deaths globally [2,3], and the prevalence of preeclampsia globally is 4.6% [2,4]. Preeclampsia has been subgrouped by the time of occurrence as early-onset pre-eclampsia (EOP) when delivery occurs before 34 weeks of gestation or late-onset pre-eclampsia (LOP) with delivery at or after 34 weeks [5]. Both forms represent subtypes of pre-eclampsia in which the causes and timing of the onset of the placental dysfunction that triggers the maternal vascular response are different. It has also been suggested that early and late-onset preeclampsia are two different diseases due to different risk factors and clinical pictures [6,7].

According to current knowledge, preeclampsia (ICD-10 code 014) is defined as a complication of pregnancy characterized by a complex of symptoms, including maternal hypertension and proteinuria, with or without pathological edema. Preeclampsia is the most dangerous complication during pregnancy because of hypertrophy and premature detachment of the placenta and intrauterine fetal death [8]. Furthermore, preeclampsia appearing after the 20th week of gestation could be connected with fetal growth restriction, maternal endothelial dysfunction and chronic immune activation [9,10]. The placental mechanism ofPE development involves two stages.The first stage is related to the changes taking place in the placenta (weak invasion of trophoblasts, incomplete vascular formation of the spiral arteries),leading to its dysfunction in early pregnancy.In the second stage, the dysfunctional placenta leads to the release of factors into the mother’s blood, which in turn leads to hypertension and organ damage [11].

Despite the fact that the cellular and tissue-specific pathways during pregnancy-induced hypertension are becoming more and more known, including vascular endothelial growth factor A (VEGF-A), mRNAs encoding VEGF-C, placental growth factor (PlGF), the angiopoietins angiopoietin 1 (Ang1) and Ang2, and the receptors VEGFR-3 (Flt-4), Tie 1 and Tie 2 [12,13], the exact etiology remains unclear.

It is clear that the placenta plays a key role in the pathology of preeclampsia [14]. Studies have revealed placental abnormalities in preeclampsia, and the unique feature of the placenta proposed to result in preeclampsia is its exposure to reduced placental perfusion [14]. Moreover, a disrupted immune system might be a predisposing factor or result of placental oxidative stress or excessive inflammation in preeclampsia [9,15,16].

On the other hand, it is well known that one of the most important chemical compounds in pregnancy is cholesterol, because carefully balanced regulation of cholesterol metabolism appears to be of critical importance for the development of the fetus [17,18]. Cholesterol is used for the synthesis of placental steroids, and an increase in the concentration of cholesterol during pregnancy causes the accumulation of maternal fat stores in the first two-thirds of pregnancy to serve as a source of calories for the mother and developing fetus [19,20]. There is known that cholesterol flow from maternal to fetal circulation is possible through the uptake of fractions such as LDL and HDL by syncytiotrophoblast and cholesterol efflux [21,22].

However, changes inthe lipid profile in the serum of preeclamptic women, such as hyperlipidemia, have also been observed [10]. A meta-analysis of seventy-four clinical studies [18] revealed that preeclampsia is associated with higher levels of total cholesterol, non-HDL-C and triglycerides, and with a diminished level of HDL-C in the third trimester. Meanwhile, the level of LDL-C was observed to be marginally associated with preeclampsia. Additionally, the increased synthesis of free radicals leads to an increase inoxidative derivatives of LDL (oxLDL) [23,24]. Mechanisms of transport of maternal cholesterol to the fetal circulation are well known [18,20,25]. 

Several ATP-binding cassette (ABC) transporter subfamilies, i.e., B, Cand G, including MDR1/P-glycoprotein, the MRPs and BCRP, are expressed in the human placenta, where they play a role in the transport of endogenous compounds and may protect the fetus from exogenous compounds, e.g., therapeutic medicines, narcotics and other xenobiotics [26,27]. In the placenta, the activity of transporter proteins from subfamilies ABCA, ABCB, ABCC and ABCG has been shown. The protein ABCA1 (adenosine triphosphate-binding cassette transporter A1) mediates the efflux of cholesterol and other phospholipids, actively influencing the changes inthe lipid profile [28,29,30] and participating in the construction of the HDL molecule [31]. Expression of the ABCA1 transporter is also high in the placenta, where it occurs in the villous cytotrophoblasts, on the surface of the syncytiotrophoblast membrane and in placental endothelial cells [26,32,33]. In the human placenta, the ABCA1 transporter plays a pivotal role, probably not only in cholesterol metabolism [34] but also inthe phospholipids, sphingomyelin, phosphatidylcholine and phosphatidylserine [26,35]. Recently, it was shown that ABCA1 expression can be linked to disorders associated with abnormal placentation, such as preeclampsia [26,36]. Studies suggest lower activity of the ABC transporter in the inflammatory process and oxidative and metabolic stress, which play a central role in the etiology of PE and gestational diabetes [37]. Moreover, the low expression of *ABCA1* mRNA correlates with an increased risk of preeclampsia and other pregnancy complications [33,36].

On the other hand, liver X receptors (LXRs) are important in this biochemical aspect because these nuclear receptors are key regulators of macrophage function, controlling transcriptional programs involved in lipid homeostasis and inflammation. When activated, LXRs trigger a series of genes that are involved in cholesterol management. LXRs have the following two isoforms: LXRA (nuclear receptor subfamily 1, group H, member 3, NR1H3) and LXRB (nuclear receptor subfamily 1, group H, member 2, NR1H2). *LXRA* is highly expressed in adipose tissue, the liver and macrophages, whereas *LXRB* is expressed in all examined tissues.Both LXRs are activated by oxysterols, oxidized derivatives of cholesterol [38,39,40].

The molecular mechanism of association of the preeclampsia with hyperlipidemia is not well understood.Therefore, the purpose of our study was to determine the mRNA and protein expression of the ABCA1 and LXRA/LXRB genes in the placenta of late-onset preeclamptic women compared to normotensive, healthy Polish women with uncomplicated pregnancies.

## 2. Materials and Methods

### 2.1. Patients

Placental tissue was obtained from 55 women who gave birth at the Division of Perinatology and Women’s Diseases, Poznan University of Medical Sciences. Sixteen placentas from preeclamptic women and 39 normal placentas from healthy pregnant women were included in the analysis. Immediately following delivery of the placenta, 3 placental tissue samples approximately 1 cm^3^ in size were removed from the maternal side in order to obtain villous cytotrophoblasts and decidua (central and marginal parts of placental disc). Following removal of the maternal and fetal surfaces, the sample was washed twice in cold PBS (phosphate-buffered saline), placed in liquid nitrogen and transported to the laboratory for storage at −80 °C.

To reduce confounding by differences in gestational age, we included in our study only late-onset preeclampsia (LOPE) women with delivery at or after 34 weeks. The patients met the criteria according to the American College of Obstetricians and Gynecologists [41] (disease occurrence after 20 weeks of gestation, systolic blood pressure 160 mmHg or more, diastolic blood pressure 110 mmHg or more, possibly proteinuria presence). The exclusion criteria were as follows: age younger than 19 or older than 35, habitual smokers, multifetal pregnancy, chronic hypertension, endocrinological diseases, kidney or liver diseases, diabetes, obesity (BMI before pregnancy ≥30), excessive weight gain in pregnancy according to Institute of Medicine 2009 Gestational Weight Gain Guideline Knowledge or even cholesterol problems. Thirty-nine healthy pregnant, normotensive women were enrolled in the control group. The patients were Caucasians of Polish origin recruited in the Division of Perinatology and Women’s Diseases, Poznan University of Medical Sciences. The Local Bioethical Committee at Poznan University of Medical Sciences approved the study. All patients were informed about the aim of the study and gave their written consent.

### 2.2. RNA Extraction and cDNA Synthesis

**Figure 1:**
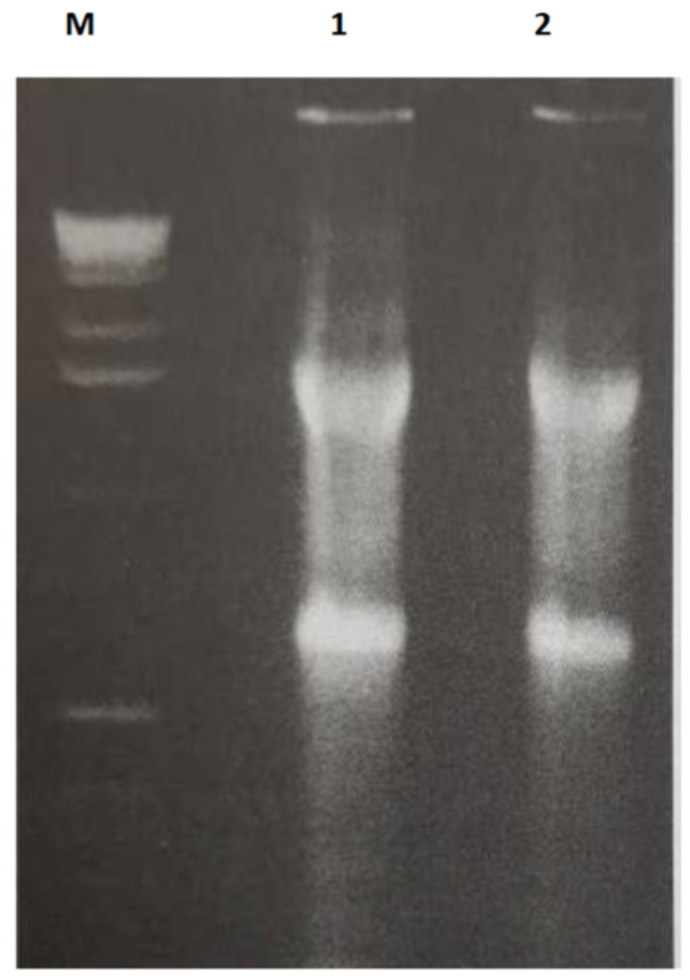
Electrophoretic separation of intact total RNA on a 1.5% denaturing agarose gel. M–1KB DNA Ladder, 1,2–RNA samples. The 18S and 28S ribosomal RNA bands are clearly visible in the intact RNA sample.

Total cellular RNA was isolated from the placental tissue using TriPure Isolation Reagent (Roche, Mannheim, Germany) according to the manufacturer’s protocol. Prior to RNA and protein isolation, 3 placental tissue samples for each patient were homogenized in one tube. The concentrations and purity of RNA were determined using a NanoDrop spectrophotometer (Thermo Fisher Scientific, Waltham, MA, USA). RNA quality was also assessed by electrophoretic separationon a 1.5% denaturing agarose gel. The 18S and 28S ribosomal RNA bands were clearly visible in the intact RNA sample (Figure 1).

RNA samples were stored at −80 °C. Complementary DNA was synthesized from 2 µg of total RNA in a total volume of 20 µL using the Transcriptor First Strand cDNA Synthesis Kit (Roche, Mannheim, Germany). The obtained transcripts were stored at −20 °C or used directly for the real-time quantitative polymerase chain reaction (RT-qPCR).

### 2.3. Real-Time qPCR

The level of mRNA expression was analyzed using theRT-qPCR method. The primers used for ABCA1, LXRA and LXRB amplifications were described in Table 1. All oligonucleotide sequences were synthesized by TIB Molbiol (Berlin, Germany). Amplicon size and reaction specificity were confirmed by agarose gel electrophoresis and melting curve analysis. RT-qPCR was carried out using a LightCycler96 Instrument (Roche, Manheim, Germany) and a LightCycler96 SYBR Green I Master (Roche, Manheim, Germany) according to the manufacturer’s protocol. *GAPDH* was used as a housekeeping gene for normalization (endogenous internal standard). The PCR program was initiated with activation at 95 °C for 10 min. Each PCR cycle comprised a denaturation step at 95 °C, an annealing step at a specific temperature and an extension step at 72 °C. The quantitative PCR was monitored by measuring the increase in fluorescence by the binding of SYBR Green I dye to the generated double-stranded cDNA. All samples were run in duplicate using the LightCycler96 Instrument and the melting curves were analyzed using the LightCycler96 Basic Software. The standard deviation between duplicates did not exceed 0.5. The resulting cycle threshold (Ct) values from the RT-qPCR were transformed into relative quantities (RQs) and normalized RQs as described by Hellemans et al. [42], assuming 100% amplification efficiency.

### 2.4. Enzyme-Linked Immunosorbent Assay

The Human ATP Binding Cassette Transporter A1 (ABCA1) ELISA Kit (sensitivity: 0.1 ng/mL; Abbexa, Cologne, Germany), Human Liver X Receptor Alpha (LXRA) ELISA Kit (sensitivity: <0.056 ng/mL; MyBioSource, San Diego, CA, USA), and Human Oxysterols receptor LXR beta ELISA Kit (sensitivity: <0.06 ng/mL; Abbexa, Cologne, Germany) were employed to evaluate the concentrations of ABCA1, LXRA and LXRB from tissue homogenates according to the manufacturer’s protocols. The absorbance was measured on a microplate reader (Infinite 200, TECAN, Männedorf, Switzerland). The concentrations were determined by interpolation of the standard curve using linear regression analysis. The protein concentration in the samples was determined by comparing the OD values of the samples to the standard curve. The standard used to create the standard curve was expressed in ng/mL.

### 2.5. Statistical Analysis

The R statistical software version 4.1.2 (R Foundation for Statistical Computing, Vienna, Austria, accessed on 12 December 2021) was used for statistical analysis and data plotting [44] and the ggstatsplot package [45]. The normal distribution of the data was tested using the Shapiro-Wilk test. Quantitative variables with Gaussian distribution were expressed as means ± standard deviation (SD), and in the absence of normal distribution as median and interquartile range (IQR). Clinical characteristics between groups were compared using Student’s *t*-test for normally distributed data and Fisher’s test for nominal variables. The Mann–Whitney U test was used for nonparametric genes and protein expression data. Associations between the expression of gene and protein pairs were assessed using the nonparametric Spearman rank correlation test with Holm-Bonferroni correction. Bivariate and multivariate logistic regression analyses were performed to calculate the odds ratio (OR) and adjusted odds ratio (AOR) with 95 percent confidence intervals (CI) were used to estimate the risk of preeclampsia. A *p*-value less than 0.05 was considered to indicate statistical significance.

## 3. Results

### 3.1. Subject Characteristics

The characteristics of the study subjects according to the presence or absence of preeclampsia are presented in Table 2. No significant differences were observed between maternal age, gestational age at delivery, height, weight and BMI before and at the end of pregnancy. Distributions of neonatal gender (50.0% vs. 51.3% of sons in the control group) did not significantly differ between the two groups. Statistically significant differences between the two groups of investigated women were found for systolic and diastolic blood pressure. For neonates, the statistically significant differences at placenta weight (385.00 ± 173.13 g vs. 556.82 ± 88.14 g in controls; *p* = 0.0014), neonatal birth weight (1921.25 ± 869.59 g vs. 3472.82 ± 457.83 in controls; *p* < 0.0001) and median Apgar scores have been noted. Blood chemistry tests were performed only in the PE group.

### 3.2. Placental Expression of LXRA, LXRB and ABCA1

The most interesting result to note was the lower expression of *LXRB* mRNA in the placentas of late preeclamptic women compared to expression in healthy women, without statistical significance (0.824 vs. 1.468 in controls, *p* = 0.0625). All results are presented in Table 3.

### 3.3. Proteins Concentration in the Placentas

**Figure 2:**
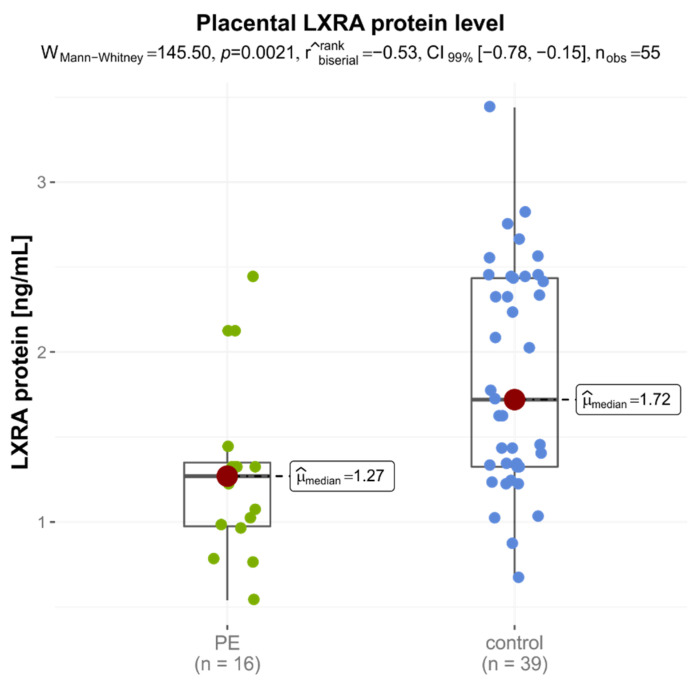
Protein expression level of LXRA in placentas of women with preeclampsia and healthy pregnant women.

Analyses of *LXRA* protein in placentas of the late-onset PE group were significantly lower than that of the control group (medians: 1.270 ng/mLvs. 1.720 ng/mL, *p* = 0.0021) (Figure 2). No significant difference was found between *LXRB* and *ABCA1* levels in placental protein levels in the two groups (Table 4).

Since 10 control patients had newborns vaginally, which could have influenced the results of the study, we performed additional analyzes to compare gene expression and protein levels between LOPE and the control group giving birth only by cesarean section (*n* = 29). The obtained results were comparable with the previous ones. We observed a statistically significant difference only for the expression of the LXRB protein in the placenta from the study and control groups (median (IQR): 1.270 ng/mL (0.975–1.350) in late-onset preeclampsia vs. 2.020 ng/mL (1.320–2.450) in 29 controls, *p* = 0.0027).

### 3.4. Correlation between Placental Protein Concentration and mRNA Expression Levels

Spearman correlation analysis was used to explore the relation between the placental LXRA, LXRB, ABCA1 genes mRNA and proteins expression in tested groups. We only found a weak, statistically insignificant positive correlation between LXRB and ABCA1 mRNA in the placenta (rho = 0.23, *p* = 0.0844). Additionally, no correlation was observed between other genes and proteins expression levels. No statistically significant correlations were found between the genes and proteins expression and various clinical mother and offspring factors (Table 5).

### 3.5. Association between Placental mRNA and Protein Expression and Late-Onset Preeclamsia

We also created logistic regression models to examine whether the relative expression levels of studied placental genes and proteins were associated with the risk of late-onset preeclampsia. Gestational age, mode of delivery, infant sex, parity and maternal pre-pregnancy BMIwere included in the multivariable model based upon their potential to confound genes and proteins expression in the placenta. As presented in Table 6, high placental LXRB mRNA and LXRA protein expression levels decreased the risk of preeclampsia, also after adjustment for possible confounders. The risk of late-onset preeclampsia incidence is associated with levels below the median of placental LXRB mRNA expression (OR = 0.26, 95% CI: 0.07–0.94, *p* = 0.040, *p* adj. = 0.018). An analogous trend was observed for placental LXRA protein levels (OR = 0.19, 95% CI: 0.05–0.69, *p* = 0.012, *p* adj. = 0.006) (Table 6).

## 4. Discussion

The placenta is an important organ with a pivotal role in the nutrition and growth of the fetus, including the supply of oxygen, energy and nutrients, removal of metabolites, as well as the synthesis of growth factors, cytokines and hormones [46]. It is arguably the most important organ in the body, but paradoxically, the most poorly understood [47]. The human placenta expresses several receptors for lipoprotein particles, but their involvement in and individual contribution to placental cholesterol uptake remains unexplored. Moreover, regulation of receptor expression under physiologic and pathologic conditions of pregnancy has been barely investigated [46].

Without a doubt, the ABC transporters play an important role in all these processes during pregnancy. It is well known that ABCA1 function is modulated by transcriptional factors such as LXRA and LXRB, which are key regulators of lipid metabolism in trophoblast cells [48]. The activation of LXRs causes an increase inthe expression of ABCA1 and ABCG1 in the placenta [46,49]. In the human placenta, both LXRA and LXRB mRNA have been identified in the early stages of gestation and can also be detected during the whole pregnancy [50]. It was reported that LXRA and LXRB mRNA expression increases in the placenta of healthy pregnant women simultaneously with the expression of typical genes for cholesterol transport. Indeed, mRNA expression is lowest in the first and second trimesters of pregnancy (25 and 24% of the term for LXRA, and 33 and 16% for LXRB, respectively). Expression levels in preterm (27–36 weeks of gestation) and term placentae (37–41 weeks of gestation) are similar to each other but significantly higher than in the first two trimesters of gestation. However, in the placenta of preeclamptic women, expression of LXRA together with ABCA1 expression can be disturbed through hypoxia in early gestation complicated by preeclampsia and in consequence influence maternal-fetal cholesterol transport [51]. The disturbances ingene expression encoding ABC transporters may elicit a disorder of lipid metabolism and may lead to dysfunction of placental function [52]. This hypothesis confirmed the results of a few studies in which changes inABC transporter expression were observed in the placenta and in the endothelial cells of the blood–brain barrier of preeclamptic women [53].

In our study carried out in a population of Polish women, we found that high placental LXRB mRNA and LXRA protein expression levels compared to the control group decreased the risk of late-onset preeclampsia, even after adjustment for possible confounders. The observation of disturbances in the expression of ABC transporters as well as LXR receptors’ mRNA in the placenta has been confirmed in only a few other studies carried out on women with PE [33,34,36,42,48,54,55], with conflicting results.

In two studies from China and Norway [42,48], the expression level of the same genes and proteins (LXRA, LXRB and ABCA1) as in our work was checked in preeclamptic placentas. Both studies established statistically significant lower levels of mRNA expression in preeclamptic placentas compared to those of the control group. Protein expressions were also significantly reduced in preeclamptic placentas as follows: ABCA1 in Chugusa et al. and LXRB in Weedon-Fekjaer et al. However, in both studies, gestational age at delivery was earlier in the preeclampsia group than in the normal pregnancy group, which could have influenced the results. In research by Weedon-Fekjaer et al., the authors point out the difficulties in retrieving placentas from premature deliveries inuncomplicated pregnancies. They note that this is ethically unacceptable and, therefore, such tissues are not available as a control group. Moreover, premature deliveries are normally due to pathological conditions (e.g., inflammation, infections or placental abruption) and are therefore not suitable as healthy controls. In our study, we compared only the placentas of the LOPE with the term-born controls [48].

In the study by Plösch et al., women with preeclampsia were divided into severe EOPE (25–33 weeks of gestation) and LOPE (34–39 weeks) and compared with respective gestational age-matched normal groups (control 1: 23–33 weeks; control 2: 34–39 weeks). They observed that the expression level of LXRA and LXRB was not significantly different between normal and preeclamptic placentas. In late-onset preeclampsia, the expression levels of LXRA, LXRB or ABCA1 were not significantly different from age-matched controls. The only expression of ABCA1 was an almost two-fold increase in EOPE, although with a notable variation. Furthermore, the authors suggested the possible regulation of LXRA and ABCA1 through placental hypoxia in preeclamptic women, which influences the transport of cholesterol [51].

Another two studies in China analyzed LXRA receptor mRNA and protein expression. The first study showed that mRNA and protein expression not only of LXRA but also of SREBP-1c wereelevated in the placentas of women with PE and increased gradually with the extent of PE among normal pregnancy, mild PE and severe PE groups. However, the mean gestational age at delivery was statistically significantly different between the PE and controls [55]. In the second study, there were no significant differences in gravidity or delivery between patients with preeclampsia and controls (*p* > 0.05). Researchers observed during a clinical trial that levels of LXRA in the serum and placenta of patients with preeclampsia were significantly higher than those in the control group, and this increase was more significant in patients with severe preeclampsia. These results were positively correlated with levels of endoglin, which is a target of LXRA in syncytiotrophoblasts [54].

Baumann et al. observed that ABCA1 mRNA expression was dependent on the gestational age and showed a significant increase in the preterm (mean ± SD: 32.8 ± 3.2 weeks) as compared to the term control (mean ± SD: 39.1 ± 0.8 weeks) placentas (*p* = 0.0013). Isolated PE, as well as PE complicated with intrauterine growth restriction (IUGR), showed a clear downregulation of ABCA1 compared to the appropriate age-matched preterm controls. However, these changes were not observed in isolated intrauterine growth restriction, HELLP syndrome (hemolysis, elevated liver enzymes and low platelets), intrahepatic cholestasis in pregnancy and gestational diabetes [33]. Moreover, Liu et al. also observed that the ABCA1 expression in the placenta and serum was lower, and the serum lipid level was higher in preeclamptic women than in healthy pregnant women. These differences correlated with PE severity [34]. On the other hand, Abrecht et al. did not observe any differences inABCA1 expression in the placenta between preeclamptic and healthy women [36].

Studies of LXRs and ABCA1 genes and proteins in human preeclamptic placentas havebeen addressed in only a few pieces of research with varying results. One of the explanations for these discrepant results isthe use of different methods (e.g., different reference genes for real-time normalization or protein expression levels studied by Western-Blot or Elisa methods). Moreover, genetic susceptibility is an important risk factor for PE. Polymorphic variant rs2695121 (T > C) of LXRB gene was found to be strongly associated with preeclampsia in (genotype CC: adjusted odds ratio, 2.05; *p* = 0.039 and genotype TC: adjusted odds ratio, 1.85; *p* = 0.049) [56]. Polymorphic variants of the genes we studied may also influence genes and proteins’ expression levels. The studies were also carried out on different populations, which may also cause differences. The authors also differently divide women with preeclampsia into subgroups. Only in one publication [51] women were subclassified according to manifestation and resolution of delivery in early- and late-onset preeclampsia.

### Strengths and Limitations

To the best of our knowledge, this is the first study of placental LXRA, LXRB and ABCA1 mRNA and protein expression in Polish women to date. A major strength is a carefully selected group of women with late-onset preeclampsia and no statistically significant differences inthe mean gestation weeks between the study groups. Participants in this study were only Caucasians of Central European ancestry (100%). We included many known risk factors for preeclampsia in our statistical analysis (gestational age, mode of delivery, infant sex, parity and maternal before pregnancy BMI) and adjusted the results for them.

There are some limitation points in our study. Firstly, we were unable to collect placental tissues from healthy women before 34 weeks of pregnancy for a control group.Moreover, we analyzed the expression of the ABCA1 transporter, whichis only one type of ATP-binding cassette transporter. It is noteworthythat, in addition to the ABCA subfamily, placental expression of other Pg-proteins (the ABCB, ABCC and ABCG subfamilies) has been demonstrated. Among the latter, expression of the ABCB1 transporter modulating placental development has been noted in the cytotrophoblast, syncytiotrophoblast and extravillous trophoblast. Moreover, it has been indicated that a decrease in ABCB1 activity in syncytial and extravillous trophoblast may play an important role in severe preeclampsia [57].

## 5. Conclusions

The lower LXRB mRNA and LXRA protein expression in the placentas ofwomen with late-onset preeclampsia suggests that these nuclear receptors could play a role in late-onset preeclampsia development through disturbances of lipid metabolism.

Liver X Receptors could be, among others, one of the elements regulating cholesterol hemostasis during pregnancy, playing a pivotal role in the PE pathomechanism by modulating blood lipid metabolism. These observations merit future studies in a larger group to identify a reliable biomarker in the prediction of preeclampsia.

## Figures and Tables

**Table 1:** Sequences of primers used in real-time quantitative PCR [43].

Gene	Forward Primer (5′-3′)	Reverse Primer (5′-3′)
ABCA1	GGAACAGGCTACTACCTGACCTTGG	ATCGATGGTCAGCGTGTCACTCTC
LXRA	GATCGAGGTGATGCTTCTGG	ACTCGAAGATGGGGTTGATG
LXRB	GATCGTGGACTTCGCTAAGCAAGTG	GTCCTTGCTGTAGGTGAAGTCCTTC
GAPDH	GAGTCAACGGATTTGGTCGTATTGG	GCCATGGGTGGAATCATATTGGAAC

ABCA1–ATP-binding cassette transporter, LXRA–liver X receptor alpha, LXRB–liver X receptor beta, GAPDH–glyceraldehyde 3-phosphate dehydrogenase.

**Table 2:** Comparison of clinical and laboratory data of mothers and newborns in two groups.

Variables	LOPE	Controls	*p*
	*n* = 16	*n* = 39	
Maternal age (years)	30.38 ± 4.50 (23–35)	30.51 ± 3.92 (20–35)	0.9101
Gestational age (weeks)	36.75 ± 1.77 (34–40)	37.54 ± 1.31 (36–41)	0.0742
Systolic blood pressure (mmHg)	175.62 ± 12.63 (160–190)	107.18 ± 9.37 (90–120)	<0.0001
Diastolic blood pressure (mmHg)	115.62 ± 6.29 (110–130)	66.54 ± 7.54 (50–80)	<0.0001
Before pregnancy BMI (kg/m^2^)	23.03 ± 4.59 (17.30–29.64)	21.59 ± 3.38 (16.73–29.75)	0.2024
After pregnancy BMI (kg/m^2^)	27.38 ± 3.43 (27.38–32.37)	26.08 ± 3.32 (19.49–33.79)	0.1992
Caesarean section, *n* (%)	16 (100.00%)	29 (74.36%)	0.0482
Primipara, *n* (%)	11 (68.75%)	12 (30.77%)	<0.0001 *
Fetal male sex, *n* (%)	8 (50.00%)	20 (51.28%)	0.9203 *
Infant birthweight (g)	1921.25 ± 869.59 (910–3940)	3472.82 ± 457.83 (2360–4440)	<0.0001
1 min Apgar score, median (IQR)	9.00 (6.50–10)	10.00 (10–10)	0.0094 ^#^
5 min Apgar score, median (IQR)	9.00 (7.75–10)	10.00 (10–10)	0.0075 ^#^
Placenta weight (g)	385.00 ± 173.13 (160–750)	556.82 ± 88.14 (400–800)	0.0014
ALT (U/L), median (IQR)	29.80 (18.60–104.70)	—	—
AST (U/L), median (IQR)	42.70 (25.90–130.20)	—	—
Urea (mg/dL), median (IQR)	36.25 (29.52–43.70)	—	—
Uremic acid (mg/dL), median (IQR)	6.18 (5.60–7.12)	—	—
Total protein (g/dL), median (IQR)	5.34 (5.14–5.76)	—	—
Creatinine (mg/dL), median (IQR)	0.84 (0.70–0.88)	—	—
Proteinuria (mg/dL), median (IQR)	500 (150–500)	—	—

AST: aspartate transaminase; ALT: alanine transaminase; values are presented as mean ± SD (min.–max.). *p*-value—Student’s *t*-test, * Fisher’s two-tailedtest. ^#^ Mann-Whitney test.

**Table 3:** Expression of mRNA *LXRA*, *LXRB* and *ABCA1* in placentas.

Gene	LOPE *n* = 16	Controls *n* = 39	*p*
*LXRA/GAPDH*	0.034 (0.008–0.106)	0.027 (0.002–0.140)	0.6234
*LXRB/GAPDH*	0.824 (0.597–1.863)	1.468 (0.756–2.762)	0.0625
*ABCA1/GAPDH*	0.983 (0.640–1.208)	1.091 (0.789–1.485)	0.1872

The values are presented as relative gene expression levels. Median (interquartile range), *p*-value calculated using the Mann–Whitney U test. ABCA1–ATP-binding cassette transporter, LXRA–liver X receptor alpha, LXRB–liver X receptor beta, GAPDH–glyceraldehyde 3-phosphate dehydrogenase.

**Table 4:** Concentrations of LXRA, LXRB and ABCA1 protein from placenta (ELISA).

Protein (ng/mL)	LOPE *n* = 16	Controls *n* = 39	*p*
LXRA	1.270 (0.975–1.350)	1.720 (1.325–2.435)	0.0021 *
LXRB	1.285 (0.785–1.570)	1.320 (0.990–1.870)	0.4925
ABCA1	2.445 (1.790–2.668)	2.220 (1.580–2.650)	0.5043

* *p* < 0.05, median (interquartile range), *p*-value calculated using the Mann–Whitney U test. ABCA1–ATP-binding cassette transporter, LXRA–liver X receptor alpha, LXRB–liver X receptor beta.

**Table 5:** Correlation coefficient matrix of placental mRNA and protein level.

Correlations	LXRA mRNA	LXRB mRNA	ABCA1 mRNA	LXRA Protein	LXRB Protein	ABCA1 Protein
LXRA mRNA	1.00	0.12	−0.15	−0.05	0.10	−0.16
LXRB mRNA	0.3970	1.00	0.23	−0.11	0.15	−0.06
ABCA1 mRNA	0.2759	0.0844	1.00	−0.08	0.21	−0.10
LXRA protein	0.7296	0.4051	0.5782	1.00	−0.18	−0.20
LXRB protein	0.4599	0.2725	0.1191	0.1987	1.00	−0.06
ABCA1 protein	0.2474	0.6714	0.4888	0.1478	0.6388	1.00

Rho–above diagonal, *p*-value below diagonal. ABCA1–ATP-binding cassette transporter, LXRA–liver X receptor alpha, LXRB–liver X receptor beta.

**Table 6:** Logistic regression models of the association between placental genes and proteins expression levels and preeclampsia.

Expression Level	≤Median >Median	Controls *n* = 39 (%)	LOPE *n* = 16 (%)	Crude OR (95% CI)	*p*	AOR (95% CI)	*p*
LXRA mRNA	≤0.033	20 (51.3)	7 (43.8)	1.00		1.00	
	>0.033	19 (48.7)	9 (56.2)	1.35 (0.42–4.36)	0.612	1.39 (0.38–5.09)	0.623
LXRB mRNA	≤1.259	17 (43.6)	12 (75.0)	1.00		1.00	
	>1.259	22 (56.4)	4 (25.0)	0.26 (0.07–0.94)	0.040	0.14 (0.02–0.82)	0.018
ABCA1 mRNA	≤1.058	18 (46.2)	9 (56.2)	1.00		1.00	
	>1.058	21 (53.8)	7 (43.8)	0.67 (0.21–2.15)	0.497	0.95 (0.23–3.92)	0.945
LXRA protein	≤1.430	14 (35.9)	12 (75.0)	1.00		1.00	
	>1.430	25 (64.1)	4 (25.0)	0.19 (0.05–0.69)	0.012	0.14 (0.03–0.63)	0.006
LXRB protein	≤1.320	17 (43.6)	8 (50.0)	1.00		1.00	
	>1.320	22 (56.4)	8 (50.0)	0.77 (0.24–2.48)	0.665	1.25 (0.31–4.95)	0.755
ABCA1 protein	≤2.320	20 (51.3)	6 (37.5)	1.00		1.00	
	>2.320	19 (48.7)	10 (62.5)	1.75 (0.53–5.77)	0.355	1.07 (0.27–4.24)	0.922

Cut-off values were medians among all study women. AOR: adjusted odds ratio; adjusted analysis corrected for gestational age, mode of delivery, infant sex, parity and maternal before pregnancy BMI, CI: confidential interval. ABCA1–ATP-binding cassette transporter, LXRA–liver X receptor alpha, LXRB–liver X receptor beta.

## Data Availability

Not applicable.
